# Immunotherapy or targeted therapy: What will be the future treatment for anaplastic thyroid carcinoma?

**DOI:** 10.3389/fonc.2023.1103147

**Published:** 2023-03-17

**Authors:** Xiaoni Gao, Chengcheng Hong, Yang Xie, Xiangtai Zeng

**Affiliations:** ^1^ Department of Thyroid and Hernia Surgery, First Affiliated Hospital of Gannan Medical University, Ganzhou, Jiangxi, China; ^2^ Ganzhou Key Laboratory of Thyroid Cancer, First Affiliated Hospital of Gannan Medical University, Ganzhou, Jiangxi, China; ^3^ Institute of Thyroid Diseases, Gannan Medical University, Ganzhou, Jiangxi, China

**Keywords:** immunotherapy, targeted therapy, anaplastic thyroid cancer (ATC), immune checkpoint blockade, MAPK, PI3K, AKT

## Abstract

Anaplastic thyroid carcinoma (ATC) is a rare and aggressive form of thyroid carcinoma (TC). Currently, there are no effective treatments for this condition. In the past few years, targeted therapy and immunotherapy have made significant progress in ATC treatment. Several common genetic mutations have been found in ATC cells, involving different molecular pathways related to tumor progression, and new therapies that act on these molecular pathways have been studied to improve the quality of life of these patients. In 2018, the FDA approved dabrafenib combined with trametinib to treat BRAF-positive ATC, confirming its therapeutic potential. At the same time, the recent emergence of immunotherapy has also attracted wide attention from researchers. While immunotherapy for ATC is still in the experimental stage, numerous studies have shown that immunotherapy is a potential therapy for ATC. In addition, it has also been found that the combination of immunotherapy and targeted therapy may enhance the anti-tumor effect of targeted therapy. In recent years, there has been some progress in the study of targeted therapy or immunotherapy combined with radiotherapy or chemotherapy, showing the prospect of combined therapy in ATC. In this review, we analyze the response mechanism and potential effects of targeted therapy, immunotherapy, and combination therapy in ATC treatment and explore the future of treatment for ATC.

## 1 Introduction

Thyroid carcinoma (TC) is one of the most common types of cancer in the world (1). Anaplastic thyroid carcinoma (ATC) contributes to more than 50% of all TC mortality, despite the fact that it accounts for only approximately 2% of all TCs (2). According to the American Thyroid Association (ATA) guidelines, the conventional treatment for ATC includes surgery, radiotherapy, and chemotherapy. Although intrathyroidal ATC who were treated with total thyroidectomy with high-dose radiation therapy and other chemotherapies show improved survival rates, prognosis is dire in patients with metastatic and progressive ATC (3). Given the poor outcomes of current therapies, the American Joint Committee on Carcinoma has categorized all ATCs as stage IV; tumors (4). In recent years, with the emergence of genome medicine and the theory of tumor immunoediting, an increasing number of clinicians have aimed to cure ATC using targeted therapy and immunotherapy.

Compared to traditional therapies, targeted therapy is more helpful in improving treatment effects and ameliorating the quality of life of patients (5). In recent years, with the rapid development of high-throughput sequencing technology, the detection of cancer-related gene mutations and the development of new targeted drugs that block related signaling pathways have allowed clinicians to flexibly adjust treatment plans. Many common gene mutations have been identified in ATC, such as BRAF, RAS, and P53 mutations (6, 7). Many signaling pathways can be targeted, such as the RAS/RAF/ERK pathway, PI3K/AKT/mTOR pathway, etc. (8) At present, many drugs targeting the above mutations and pathways have been developed to carry out a large number of clinical trials showing various results.

Thanks to the pioneering work of the 2018 Nobel Prize winners in medicine, James Patrick Allison and Tasuku Honjo, immune checkpoints and immunotherapy have become popular topics. Currently, immunotherapy is used in the clinical treatment of patients with various cancers, such as non-small cell lung cancer and melanoma, and has achieved good therapeutic effects (9, 10). Immunotherapy, especially immune checkpoint blocking therapy, has also made good progress in the treatment of ATC. Currently, immunotherapy is considered a promising strategy by some experts, and some related drugs for ATC are being researched clinically, but there are still many patients who do not respond to immunotherapy or develop treatment resistance that needs to be addressed (11). Simultaneously, scientists have been pleased to discover that immunotherapy drugs combined with targeted therapy drugs can increase the anti-tumor effect of targeted therapy drugs (12).

In this article, we review all the clinical studies on targeted therapy and immunotherapy for ATC, looking for the best methods of combining targeted therapies and immunotherapy.

## 2 Targeted therapy

**Figure 1:**
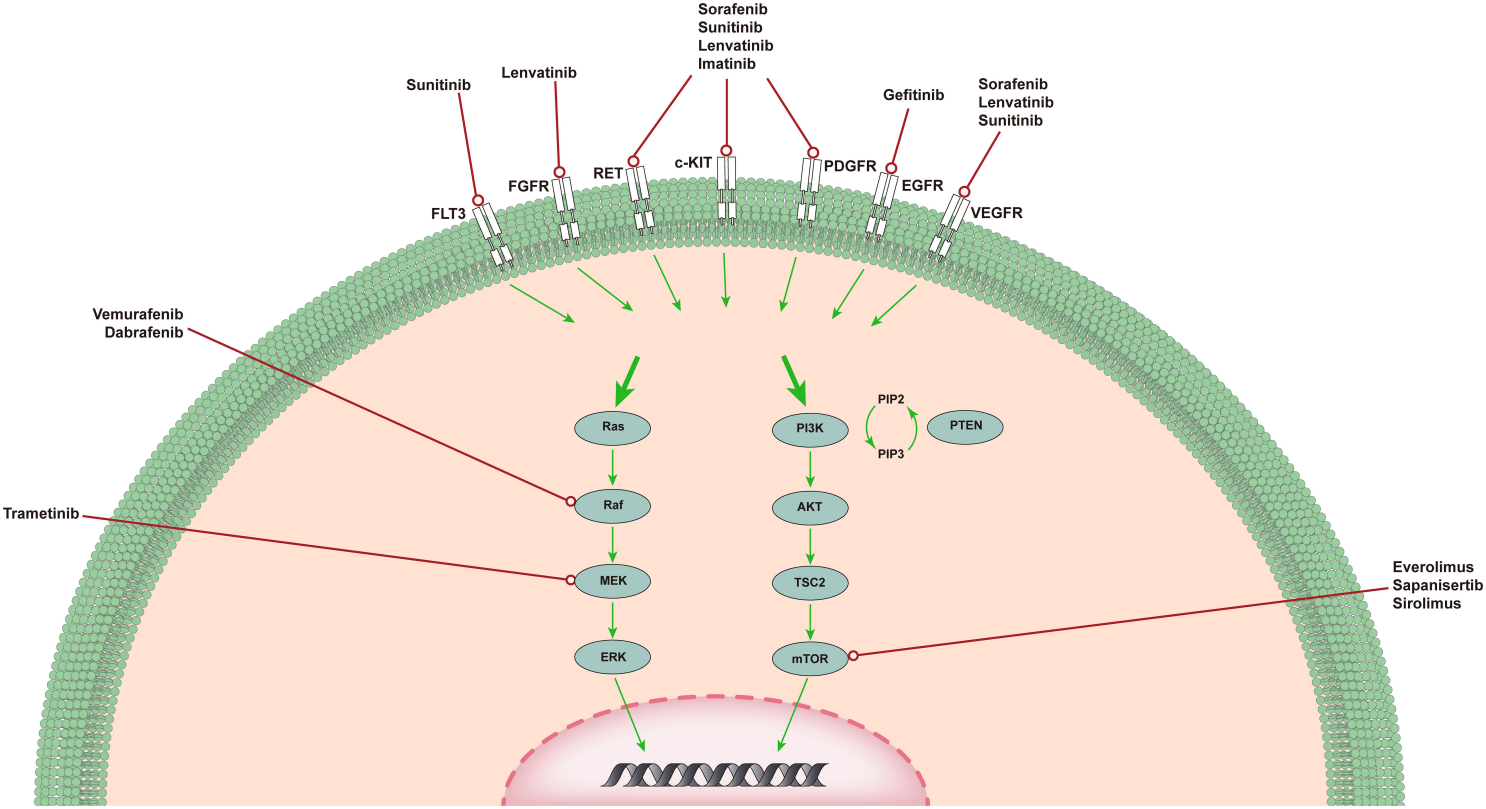
The MAPK pathway and PI3K/AKT/mTOR pathway in ATC. MAPK and PI3K/AKT pathways in ATC. The MAPK and PI3K/AKT pathways are responsible for angiogenesis, proliferation, and tumorigenesis. Shown are several medications capable of specific mutations, targeting upstream receptor tyrosine kinases, and genetic rearrangements that have completed or are in clinical trials of targeted therapy for ATC.

Targeted therapy usually refers to treatments that specifically target molecules related to the tumor formation process and cause less damage to normal cells. Targeted therapies block the activity of specific molecules that are essential for cancer growth and development (13). Most targeted therapies include small-molecule or monoclonal antibodies (14). Here, we present the most common out-of-control signaling pathways in ATC (**Figure 1**) and outline the corresponding drugs and their relevant advances in clinical experiments.

### 2.1 MAPK pathway

The MAPK signaling pathway plays an important role in the occurrence and development of ATC (15). Signal transduction through the MAPK pathway occurs after extracellular growth factor binds to a variety of tyrosine kinase receptors (TKRs), which in turn leads to the activation of RAS (16). RAS is a small GTP-binding protein that exists in three isotypes: HRAS, KRAS, and NRAS. After activation, NRAS binds BRAF to phosphorylate and activate MEK (17). MEK sends a signal to ERK, which enters the nucleus and enhances the transcription of a number of transcription factors, leading to increased cell proliferation and cell survival. Since the discovery of this signaling pathway, researchers have gradually developed drugs that target this pathway for testing, such as tyrosine kinase inhibitors (TKIs), BRAF inhibitors, and MEK inhibitors.

#### 2.1.1 Tyrosine kinase inhibitors

At present, it has been found that TKI can achieve its anti-tumor purpose by inhibiting the repair of tumor cells, blocking cell division in the G1 phase, inducing and maintaining cell apoptosis, and inhibiting angiogenesis (5). Some phase 1 and phase 2 clinical trials have reported the results of tyrosine kinase inhibitor monotherapy for ATC, and the proportion of patients who achieved objective remission was between 0% and 25% (18–24).

Sorafenib is the first oral multi-kinase inhibitor that targets BRAF, rearranged during transfection (RET), KIT, platelet-derived growth factor receptor (PDGFR), and vascular endothelial growth factor receptor (VEGFR) 1-3. The NCT00126568 trial evaluated the effectiveness of sorafenib in anaplastic thyroid cancer (18). Among the 20 recruited patients, the disease control rate (DCR) was 35%, and the median overall survival (OS) was 3.9 months. In this experiment, patients who used sorafenib only experienced grade 3 and grade 4 toxic reactions with mild symptoms. In contrast, the NCT02114658 trial evaluated the safety and effectiveness of sorafenib in Japanese medullary thyroid carcinoma (MTC) and ATC patients and concluded that it seems to be effective for advanced MTC but ineffective for ATC (19). A total of 20 patients were recruited in this study, of whom 8 had MTC and 10 had ATC. Two of the MTC patients had a partial response (PR) (25%), but none of the ATC patients had symptom relief, and only four patients showed stable disease (SD) (40%). To further explore the role of sorafenib in the treatment of ATC, a study on sorafenib as an adjuvant treatment for ATC was carried out (NCT03565536). It was expected that 10 participants would participate in the trial. This trial used sorafenib as adjuvant treatment and surgery if the patient’s condition was relieved.

Lenvatinib is a representative oral TKIs, targeting VEGFR1-3, fibroblast growth factor receptor (FGFR) 1-4, PDGFR, RET, and KIT. A phase II trial tested the effectiveness and safety of lenvatinib for the treatment of patients with advanced thyroid cancer (20). Of the 51 patients, 17 with ATC were recruited, and most of them received chemotherapy and radiotherapy. Among them, 4 patients achieved remission (24%). This high-response effect in ATC patients is encouraging, and the toxicity is controllable, but the study did not evaluate the correlation between biomarkers and the efficacy of lenvatinib in ATC. Therefore, lenvatinib will be examined as an exploratory endpoint in a phase 2 trial of patients with ATC (NCT02657369, NCT02726503). The current NCT02657369 trial was terminated because there was only one PR (3%) among the 33 recruited patients. The results of the NCT02726503 trial have not yet been reported.

In addition to the TKIs mentioned above, other TKIs have also undergone clinical research. Imatinib is an inhibitor of Bcr/Abl, PDGFR, c-Fms, KIT, and RET. A clinical study on imatinib in advanced ATC demonstrated its efficacy and good tolerability (23). Eleven patients with ATC were recruited in this study. Eight of the 11 patients had evaluable treatment effects. Two patients (25%) developed PR, and four patients (50%) developed SD. Sunitinib is an oral multitargeted TKI that inhibits VEGFR1-2, PDGFR, KIT, FMS-like tyrosine kinase-3 (FLT3), receptor of macrophage-colony stimulating factor (CSF1R), and RET. An article evaluated the efficacy and safety of sunitinib in the treatment of locally advanced or metastatic TC (NCT00510640) (22). The study recruited a total of four ATC patients, and the results of the study showed that only one ATC patient achieved SD. Compared with the other two TC subtypes in this study, sunitinib did not change the prognosis of patients with ATC. Gefitinib is an EGFR inhibitor, and a phase II clinical study on gefitinib for the treatment of advanced TC evaluated its efficacy (24). Among the 5 ATC patients recruited in this study, only 1 patient with ATC achieved SD. Pazopanib is a VEGFR, PDGFR, and KIT inhibitor. A phase II study (n=16) of patients with advanced ATC evaluated pazopanib, but one patient withdrew before the start of treatment (21). Although transient disease regression was observed in several patients, no RECIST responses were confirmed. It cannot be proven that pazopanib monotherapy is a reasonable treatment for ATC.

In general, these results indicate that the above-mentioned TKIs show moderate single-agent activity in the treatment of ATC. Some clinical trials show that patients may not respond well to the drug because of the small number of patients; therefore, it may be necessary to further study the application of TKI in ATC in a multi-institutional environment.

#### 2.1.2 BRAF inhibitor

BRAF is one of the first and most studied point mutations in thyroid cancer (25). BRAF is part of the MAPK pathway and is essential for the regulation of cell growth, proliferation, and survival (26). BRAF mutations have been observed in 41% of ATC patients; therefore, targeting BRAF is very important for the prognosis of ATC patients (27). Kristen et al. found that a 51-year-old male patient was diagnosed with ATC, and genetic analysis showed that BRAF had a mutation (28). Subsequently, treatment with the BRAF inhibitor, vemurafenib, was initiated. The patient experienced progressive dyspnea. Comuted tomography (CT) of the chest showed lung infiltration and nodule deterioration. On day 38, 18F-FDG-PET and CT of the chest indicated that the metastatic disease was almost completely eliminated. A phase II clinical study evaluated the role of vemurafenib in a variety of non-melanoma cancers with BRAFV600 mutations (NCT01524978) (29). Among the seven recruited patients with ATC, there was 1 complete response (CR) and 1 PR. Although the number of patients recruited was small, this is notable because of the limited treatment options available for ATC. Furthermore, dabrafenib has also shown antitumor activity in patients with BRAFV600E mutant anaplastic thyroid cancer. However, dabrafenib is generally used in combination with other drugs because of the resistance of thyroid cancer cells.

### 2.2 PI3K/AKT pathway

The PI3K/AKT pathway is the second most frequently dysregulated pathway after the MAPK pathway in ATC (30). Similar to MAPK, the PI3K cascade is triggered by the RTK and RAS proteins. Once activated by NRAS, PI3K catalyzes the phosphorylation of PIP2 to PIP3. PIP3 acts as a second messenger, and its production is inhibited by PTEN. PIP3 can activate AKT, which in turn phosphorylates mTOR and a number of other targets, so that cancer develops in a direction that is more conducive to its survival. As a downstream molecule of the PI3K/AKT pathway, mTOR signaling is commonly activated in tumors and controls cancer cell metabolism by altering expression and/or activity of a number of key metabolic enzymes (31). One research showed that mTORC1 signaling affects one-carbon metabolism through an ATF4-dependent transcriptional induction of the mitochondrial tetrahydrofolate cycle (32). In addition, a previously uncharacterized protein, SAMTOR, could function as a SAM sensor linking one-carbon metabolism to mTORC1 signaling (33). Currently, related inhibitors of mTOR have been clinically tested in patients with ATC.

As early as 2013, a study on the efficacy and safety of the mTOR inhibitor everolimus in the treatment of locally advanced or metastatic TC (NCT01164176) evaluated the therapeutic effect of everolimus on ATC (34). Among the six recruited TC patients, none of the ATC patients experienced remission. In a subsequent study to determine the efficacy and safety of everolimus in patients with advanced follicular TC, 7 patients with ATC were enrolled (35). However, in the entire study, no ATC patients benefited. Fortunately, in a clinical phase II study on the efficacy of everolimus in radioiodine-refractory TC, among the seven ATC patients recruited, one ATC patient (14%) exhibited PR. Two patients (28%) had a median progression-free survival (PFS) of 2.2 months (36). The study also performed genetic sequencing of six patients with ATC. Preliminary data show that patients with ATC with mTOR mutants exhibit the greatest benefit.

Sapanisertib (MLN0128) is a new type of mTOR inhibitor that has previously been shown to have anti-tumor activity in other cancer patients (37). Currently, a phase II clinical trial of sapanisertib (MLN0128) for the treatment of metastatic ATC is enrolling patients (NCT02244463) and is expected to be completed before December 2022.

### 2.3 Combined targeted drugs

The diversification of tumor occurrence and development, as well as intertwined regulatory mechanisms, have brought challenges to targeted therapies. However, because of mutations in different targets in tumors and the intertwined regulatory mechanisms between signaling pathways, there may be the possibility of combined therapy between different targeted drugs. We considered BRAF inhibitors as an example. At present, TC and other cancer cells mainly prevent BRAF inhibitors from exerting their effects by reactivating the drug resistance mechanism mediated by the MAPK pathway (38). This mainly includes increasing the expression of RTK, activating mutations in upstream signals and changes in downstream MAPK pathways, activation of parallel signaling pathways, BRAF amplification, and alternative splicing. Therefore, completely blocking the MAPK pathway may be necessary to enhance the anti-tumor activity of BRAF inhibitors. In one study, the combination of the MEK inhibitor PD0325901 plus PLX4720 resulted in a better inhibitory effect on ATC cell growth than PLX4720 alone (BRAF inhibitor) (39). Moreover, one study reported that the combination of trametinib and pazopanib in anaplastic thyroid cancer cell lines resulted in synergistic inhibition of tumor growth (40). This shows that combined targeted therapy provides new possibilities for the prognosis of patients with ATC, and the current combined targeted therapy method has been used in clinical practice.

Previously, a phase II clinical study evaluated the efficacy and safety of dabrafenib plus trametinib in patients with locally advanced or metastatic BRAF V600 mutant ATC (NCT02034110) (41). At the beginning of the study, 16 ATC patients were sequenced, and 15 ATC patients were found to have BRAF mutations. Preliminary results showed that 11 patients were in remission (1 CR and 10 PR). Because dabrafenib plus trametinib has strong clinical activity against BRAF V600E mutant ATC, it has been approved by the FDA for the treatment of patients with BRAF-positive ATC. In addition, another phase I study is currently underway. This study aimed to evaluate the efficacy of dabrafenib, trametinib, and intensity-modulated radiation therapy (IMRT) (NCT03975231). The study will be conducted on 6 patients and is expected to be completed in April, 2025. There have also been trials on other targeted drug combination therapies. A phase II clinical study of radioiodine-refractory TC evaluated the efficacy of the combination therapy of the mTOR inhibitor sirolimus and sorafenib (42). Among the two recruited patients with ATC, one patient had PR and did not carry the BRAF mutation.

The above studies have shown that targeted therapeutic agents can achieve potential therapeutic effects in ATC. However, to date, the experimental results for most targeted therapies have been unsatisfactory, and resistance to kinase inhibitors remains a major obstacle in ATC therapy. Furthermore, several clinical trials are underway to explore the appropriate timing and sequence of targeted therapy in ATC (**Table 1**), and more comprehensive conclusions can be drawn regarding the effects of targeted drugs on ATC.

**Table 1:** Ongoing clinical trials of targeted therapy for ATC.

Drugs	NCT number	Study phase	Status	Description	Estimated enrollment	Primary completion date
MLN0128	02244463	II phase	Active, not recruiting	This research study is studying a targeted therapy (ML0N128) as a possible treatment for anaplastic thyroid cancer. In this research study, the investigators are investigating usefulness of MLN0128 in metastatic anaplastic thyroid cancer cases.	46	December 2022
Sorafenib	03565536	II phase	Unknown	This study attempted to apply it to preoperative treatment of undifferentiated cancer to see if it would shrink the tumor and give the patient an opportunity for surgery.	10	April 30, 2019
Dabrafenib and Trametinib	03975231	I phase	Recruiting	This trial studies how well dabrafenib, trametinib, and intensity modulated radiation therapy (IMRT) work together in treating patients with BRAF mutated anaplastic thyroid cancer.	20	April 30, 2025

Source: https://clinicaltrials.gov/.

## 3 Immunotherapy

**Figure 2:**
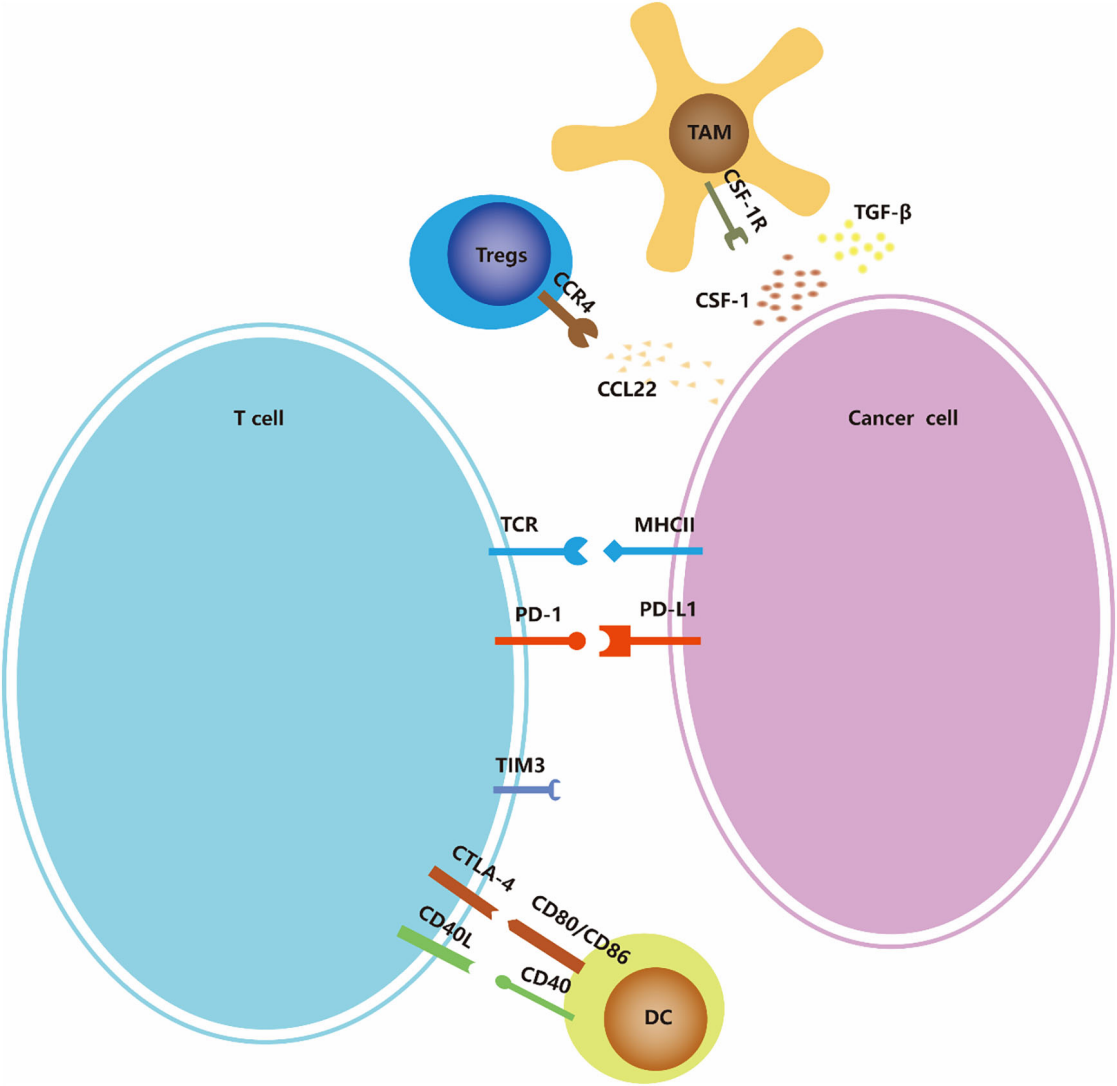
Immune-checkpoints and co-stimulatory signaling. Many of the ligands bind to multiple receptors, some of which deliver co-stimulatory signals and others deliver inhibitory signals. The expression of ligands and receptors should not be considered exhaustive; for example, PDL1/PDL2 is also expressed by antigen-presenting cells such as macrophages and dendritic cells (not shown in the figure), and MHC-II is also expressed by dendritic cells.

Cancer immunotherapy is cancer treatment that induces, enhances, or inhibits specific immune responses. It involves multiple immune cells, including overcoming immune suppressive signaling, T cell initiation and differentiation, and enhancing tumor-associated antigen (TAA) presentation. (**Figure 2**) In addition to the above-mentioned tumors that can change proliferation and utilize other methods to resist attack by the immune system, tumors can also escape immune surveillance by immune editing (43). Next, we introduce several immunotherapy strategies used for ATC, including immune checkpoints, adoptive cell therapy, and oncolytic viruses as three aspects to focus on.

### 3.1 Immune checkpoint blockade

The immune checkpoint refers to a series of immunosuppressive pathways of immune cells that regulate and control the persistence of the immune response while maintaining self tolerance (44). At present, researchers have conducted many studies on PD-1/PD-L1, but research on other immune checkpoints, such as CTLA-4 and CD27, is still rare in ATC.

#### 3.1.1 CTLA-4 blockade

Cytotoxic T lymphocyte-associated antigen-4 (CTLA-4), also known as CD152, is a transmembrane receptor expressed on T cells. Its ligands and CD28’s are B7 molecules; that is, the costimulatory molecule CD80/CD86 (also known as B7-1/B7-2) is expressed on the surface of antigen presenting cells (APCs) (45). The binding affinity of CTLA-4 to B7 is much higher than that of CD28 (46, 47). When T cells are activated, CTLA-4 is upregulated and competes with CD28 to bind to B7, thereby transmitting the inhibitory signal of T cell activation and participating in the negative regulation of the immune response (45). One study found that the CD80 mRNA levels decreased in 81.82% (9/11) of ATC patients (48).

Anti-CTLA-4 drugs play a role in the initiation of the immune response by inhibiting the interaction between CTLA-4 on T cells and B7 on APCs. Ipilimumab is a human monoclonal antibody, IgG1, that inhibits the interaction between CTLA-4 and its ligand. In 2011, as a result of the improvement in clinical efficacy, the FDA approved ipilimumab for the treatment of unresectable stage III/IV melanoma. The study showed that compared with placebo, the relapse-free survival (RFS), overall survival (OS), and distant metastases-free survival of melanoma patients treated with ipilimumab were significantly better (49). One study confirmed that the CTLA-4 ligand in papillary thyroid carcinoma (PTC) and ATC tissues is deregulated as PD-1, suggesting the possible prognostic value of CD80 gene expression in ATC (48). Currently, a clinical trial of CTLA-4 antagonists combined with PD-1 antagonists for ATC is being conducted (NCT03246958).

#### 3.1.2 PD-1/PD-L1 blockade

PD-1 (programmed death receptor 1) is an inhibitory receptor expressed on a variety of immune cells including T cells, B cells, dendritic cells (DCs), monocytes, and natural killer cell (NK) receptors (50). The interaction between PD-1 and its ligand PD-L1 or PD-L2 leads to the downregulation of effector T cell responses and mediates immune tolerance, resulting in the immune escape of tumor cells.

PD-L1 is overexpressed in many types of tumors and is associated with poor prognosis. Many studies have found that PD-L1 is highly expressed in ATC tissues and can promote tumor cells (48, 51–56). Lymphocyte infiltration in the ATC group was significantly higher than that in the differentiated thyroid carcinoma (DTC) group, and PD-L1- or PD-1-positive lymphocytes were significantly higher than those in the DTC group (57). High PD-1/PD-L1 expression predicts poor prognosis in patients with ATC in terms of OS and progression-free survival (PFS) (53). This indicated that the PD-1/PD-L1 pathway plays a key role in ATC.

Currently, research on PD-1/PD-L1 inhibitors has been successful. Spartalizumab (PDR001) is a humanized monoclonal antibody that targets PD-1 on the surface of human immune cells with immune checkpoint inhibition and anti-tumor activity (58). In a phase II clinical trial (59), patients with locally advanced and/or metastatic ATC were intravenously injected with 400 mg of spartalizumab every 4 weeks. The overall remission rate (ORR) was 19%, including 3 patients with complete remission and 5 patients with partial remission, which confirmed the efficacy of spartalizumab (PDR001), a PD-1 inhibitor, in the treatment of ATC. It can be seen that spartalizumab has good clinical activity and safety in patients with malignant incurable diseases and short life span. In addition, a single-arm and multi-center study using PD-1 monoclonal antibody HX008 as the drug has not yet begun to be conducted, and the purpose is to evaluate the efficacy and safety of HX008 injection in patients with metastatic or locally advanced ATC (NCT04574817). Meanwhile, combination therapy with anti-PD-1 antibody and CTLA-4 in patients with stage II TC is currently ongoing (NCT03246958). To test the effectiveness of PD-L1 blocking combined therapy, other clinical trials are ongoing (NCT03181100, NCT03122496, and NCT04400474). These studies suggest that blocking the PD-1/PD-L1 pathway using checkpoint blockers may be an effective treatment. All clinical trials regarding the PD-1/PD-L1 axis block for ATC are listed in **Tables 2**, **3**.

**Table 2:** Clinical trials of immunotherapy for ATC.

Intervention	NCT number	Study phase	PD-1/PD-L1 antagonist	Status	Description	Primary completion date
PD-1 antagonist	04574817	II phase	HX008	Not yet recruiting	This is a single-arm, multicenter study to evaluate the efficacy and safety of HX008 injection in patients with metastatic or locally advanced anaplastic thyroid cancer.	May 30, 2022
PD-1 antagonist	02688608	II phase	Pembrolizumab	Completed	The purpose of this study is to assess the efficacy of pembrolizumab in patients with metastatic ATC.	October 2020
PD-1 antagonist	02404441	I/II phase	PDR001	Completed	The purpose of this study is to characterize the safety, tolerability, PK, PD, and anti-tumor activity of PDR001 administered as a single agent to adult patients with solid tumors (include ATC).	July 21, 2020
PD-1 antagonist combined with CTLA-4 antagonist	03246958	II phase	Nivolumab	Active, not recruiting	This study is evaluating nivolumab in combination with ipilimumab as a possible treatment for TC (include ATC).	December 2022

TC, thyroid carcinoma; ATC, anaplastic thyroid carcinoma; PK, pharmacokinetic; PD, pharmacodynamics. Source: https://clinicaltrials.gov/.

**Table 3:** Clinical trials of combination strategies for ATC.

Intervention	NCT number	Study phase	Drug	Status	Description	Primary completion date
PD-1 antagonist combined with chemotherapy	03211117	II phase	Docetaxel Doxorubicin Hydrochloride	Completed	This trial studies how well pembrolizumab, chemotherapy, and radiation therapy work with or without surgery in treating patients with ATC.	February 12, 2018
PD-L1 antagonist combined with chemotherapy	03181100	II phase	Atezolizumab Cobimetinib Vemurafenib Bevacizumab Nab-paclitaxel Paclitaxel	Recruiting	This trial studies how well atezolizumab in combination with chemotherapy works in treating patients with ATC or PDTC.	July 27, 2023
PD-1 antagonist combined with targeted therapy	04429542	I phase	Pembrolizumab BCA101	Recruiting	This is a Phase 1/1b, open-label study, which consists of dose escalation parts (Part A) followed by expansion cohorts (Part B) for both single agent BCA101 and combination BCA101 plus pembrolizumab in patients with EGFR-driven advanced solid tumors (include ATC).	December 31, 2023
PD-1 antagonist combined with targeted therapy	04171622	II phase	Pembrolizumab	Recruiting	This phase II trial studies how well lenvatinib and pembrolizumab work in treating patients with anaplastic thyroid cancer that is stage IVB and has spread to nearby tissue or lymph nodes (locally advanced) and cannot be removed by surgery (unresectable), or stage IVC that has spread to other places in the body (metastatic).	August 31, 2023
PD-1 antagonist combined with targeted therapy	04675710	II phase	Pembrolizumab Dabrafenib Trametinib	Recruiting	This phase II trial studies the effect of pembrolizumab, dabrafenib, and trametinib before surgery in treating patients with BRAF V600E-mutated anaplastic thyroid cancer.	June 30, 2024
PD-1 antagonist combined with targeted therapy	04731740	II phase	Pembrolizumab Lenvatinib	Suspended	The aim of the study is to evaluate the efficacy of the combination of lenvatinib with pembrolizumab, and to establish a safe and effective systemic treatment regimen for patients with metastatic anaplastic thyroid cancer (ATC)/poorly differentiated thyroid cancer (PDTC).	December 28, 2023
PD-1 antagonist combined with targeted therapy	04238624	II phase	Dabrafenib Trametinib Cemiplimab	Recruiting	This study is being done to see if adding the study drug, cemiplimab, to the standard therapy with dabrafenib and trametinib is an effective treatment against anaplastic thyroid cancer.	June 20, 2024
PD-1 antagonist combined with targeted therapy	02501096	I/II phase	Pembrolizumab Lenvatinib	Completed	Patients with solid tumors (include ATC) are currently being recruited, and the MTD of lenvatinib (E7080) combined with pembrolizumab for solid tumors will be confirmed in phase 1 B. Subsequent Phase 2 trials will assess the safety and effectiveness of the combination.	August 18, 2020
PD-L1 antagonist combined with targeted therapy	04400474	II phase	Cabozantinib Atezolizumab	Recruiting	The primary objective is to assess the efficacy of cabozantinib plus atezolizumab combination by means of radiological objective response rate (ORR) evaluated following RECIST v1.1 criteria in advanced endocrine tumors (include ATC).	March 2023
PD-L1 antagonist, CTLA-4 antagonist combined with radiotherapy	03122496	I phase	Durvalumab Tremelimumab	Completed	The purpose of this study is to test the safety of durvalumab (MEDI4736) and tremelimumab in combination with radiation therapy and find out what effects, if any, this combination has on people, and whether it improves OS.	June 3, 2022
MEK inhibitors combined with chemotherapy	03085056	I phase	Trametinib Paclitaxel	Active, not recruiting	The purpose of this study is to test the safety and tolerability of this treatment combination of paclitaxel and trametinib. Additionally, this study aims to find out what effects the combination of paclitaxel and trametinib has on the shrinkage and growth of anaplastic thyroid cancer.	September 2023
Tyrosine kinase inhibitors combined with chemotherapy	01236547	II phase	Paclitaxel Pazopanib Hydrochloride	Completed	This randomized phase II trial studies the side effects and how well intensity-modulated radiation therapy (IMRT) and paclitaxel with or without pazopanib hydrochloride works in treating patients with anaplastic thyroid cancer.	March 9, 2020

TC, thyroid carcinoma; ATC, anaplastic thyroid carcinoma; PDTC, poorly differentiated thyroid carcinoma; MTD, maximum tolerable dose; OS, overall survival; ORR, objective response rate; PK, pharmacokinetic; PD, pharmacodynamics. Source: https://clinicaltrials.gov/.

In some studies, checkpoint inhibitors were discontinued because of toxicity, but their overall tolerance was superior to that of chemotherapy. Immune-related adverse events are usually caused by autoimmune inflammation in various organs because of the excessive activation of T cells. Kolllipara et al. (60) described the abnormal reactions of BRAF-positive patients treated with PD-1 inhibitors (nivolumab) who developed nausea, vomiting, and diarrhea during the 12th cycle of nivolumab administration and were diagnosed with acute colitis by colonoscopy. Similarly, another study also described the adverse manifestations of rapid and intense response to pembrolizumab monotherapy in ATC patients (61). These results reflect the potential and serious adverse reactions associated with PD-1/PD-L1 inhibitors. In one case report (62), two patients with ATC received anti-PD-1 drug treatment, of which one had poor efficacy. This also reflects that, in some cases, ATC patients have a poor response to PD-1/PD-L1 inhibitors, and the response rate to different drugs varies greatly. Therefore, the toxicity of inhibitors and poor responses of some patients are still issues that need to be overcome.

It is worth noting that the success of anti-PD-1/PD-L1 therapy depends not only on positive PD-L1 expression but also on CD8^+^ tumor-infiltrating lymphocyte density and the ability of CD8^+^ T cells to recognize tumor antigens (55, 63). This suggests a new approach to addressing the unsatisfactory effects of anti-PD-1/PD-L1 therapy.

#### 3.1.3 Other immune checkpoint blockades

In addition, the abnormal expression of immune checkpoint molecules such as CD27, CD47, and CD70 has also been reported in ATC tissues (52, 64). Thus, these molecules may be potential targets for ATC immunotherapy.

CD27 is a member of the tumor necrosis factor (TNF) receptor family, and its ligand CD70 is a member of the tumor necrosis factor (TNF) superfamily (65). CD27 is constitutively expressed in T lymphocytes, B lymphocytes, and NK cells (66). In a study by Karen et al. (52), the expression of CD70 in 49 ATC cases was analyzed. The results showed that CD70 expression was upregulated in 49% of the samples and was diffusely expressed in 41.7% of the samples. They also noticed that CD27 expression was weak and focal in all three specimens. All ATC samples expressed CD27 in the surrounding lymphocyte subsets and were infiltrated by the tumor. These data indicate that CD27-CD70 in ATC mainly occurs in CD27^+^ lymphocytes that are in contact with CD70^+^ tumor cells. In summary, this report demonstrated that CD70 expression exists in a large number of ATC samples. CD70 can be used as an anti-tumor target for immunotherapy. Since lymphocytic infiltration of tumors is generally low, further studies are needed to determine the most effective therapy for patients with ATC.

CD47 is a quantic transmembrane receptor that inhibits phagocytosis *via* its anti-receptor signaling regulator protein α(SIRPα) (64). In another study (67), Christian et al. analyzed the expression of CD47 in 19 human primary ATC tissues. The results showed that TAMs heavily infiltrated human ATC samples, and CD47 and calreticulin were also expressed. Blocking CD47 promotes macrophage phagocytosis in ATC cell lines and inhibits tumor growth *in vitro*. To verify the validity of the *in vitro* phagocytosis experiment, anti-CD47 antibodies were used to treat immunized ATC cell line xenograft mice and tamoxifen-induced ATC double-transgenic mice. Experiments in mice showed that the treatment of ATC xenograft mice with anti-CD47 antibody increased TAM frequencies, enhanced expression of macrophage activation markers, enhanced tumor cell phagocytosis, and inhibited tumor growth. Blocking CD47 in tumor cells expressing CD47 increased TAM frequencies in double-transgenic ATC mice. These results suggest that blocking CD47 as a target could potentially improve the prognosis of patients with ATC and may be a valuable supplement to the current treatment standards.

### 3.2 Adoptive cell transfer and CAR-T cell therapy

Adoptive cell therapy is another type of immunotherapy that relies on the active, sufficient recruitment of anti-tumor T cells in the body (68). Two methods have been used: one is to inject patients with natural host cells that have been expanded *in vitro* and the other is to use chimeric antigen receptor T (CAR-T) cells to specifically recognize and kill tumor cells to treat different malignant tumors (69, 70). Several studies have confirmed that both NK cells and CAR-T cells can effectively kill ATC cells (71, 72).

NK cells are important effectors of innate immunity and play an important role in maintaining homeostasis by producing cytokines and exhibiting effective cytotoxic activity. In addition, NK cells play a key role in adaptive immune mechanisms (73). Low levels of NK cells have been reported in thyroid tumors (74). In one study (72), a retrovirus was used to transfer the Effluc gene into human NK cells (NK-92 mi), and human ATC cells (CAL-62) were transferred with Effluc and Rluc genes. ATC lung metastasis was observed by intravenous injection of CAL-62 in nude mice with lung metastasis or xenografts. Five million NK-92MI cells were injected twice into the caudal vein of nude mice. It was observed that NK cells can significantly inhibit the growth of metastatic tumors. This suggests that NK cell-based immunotherapy may be an effective treatment for ATC lung metastases.

T cells in CAR-T cell therapy have been genetically modified to express transmembrane proteins, which are synthetic T-cell receptors and antigens that target predefined tumor expression. Adoptive CAR-T cell therapy has achieved very good results in hematological malignancies (75). Research on CAR-T cells in non-hematological solid tumors is ongoing. In a breakthrough animal experiment (15), it was found that ICAM-1 CAR-T cells that target intercellular adhesion molecule-1 can mediate strong and lasting anti-tumor activity, leading to tumor eradication and a significant increase in the long-term survival of ATC xenograft mouse models. Although the expression level of ICAM-1 in some ATC cells varies, CAR-T cells can induce an increase in ICAM-1 expression so that all cells become targetable cells. ICAM-1 CAR-T cells have been used in clinical trials of patients with ATC (NCT04420754).

NK cell-based immunotherapy may be an effective treatment for ATC lung metastases. Treatment with ICAM-1 CAR-T cells has been theoretically supported, and relevant clinical experiments have also been conducted. Although both adoptive cell therapy methods are promising for ATC, their clinical significance needs to be further explored.

### 3.3 Oncolytic virus therapy

Oncolytic viruses are non-pathogenic viruses that specifically infect cancer cells. These natural or genetically engineered viruses are less toxic to normal cells but can kill cancer cells, and the release of tumor antigens by dissolution and destruction of cancer cells can stimulate the immune system and enhance immune function (76). Some studies have identified several oncolytic viruses that can effectively inhibit the proliferation of ATC cells, including dl922-947, poxviruses, and Newcastle disease virus (77–79). According to a previous study (77), dl922-947 impairs ATC-induced *in vitro* angiogenesis and monocyte chemotaxis by reducing IL-8 and CCL2 levels. In an *in vivo* ATC model, dl922-947 treatment reduced angiogenesis and TAM density. The vaccinia virus is an effective poxvirus that can effectively control proliferation and induce cell death in ATC cell lines (78). In addition, the oncolytic Newcastle disease virus (NDV) has shown potential to induce tumor cell death in a variety of cancer cells from different sources. Jiang et al. (79) found that the recombinant reporter virus rFMW/GFP showed oncolytic activity in ATC cells through the p38 MAPK signaling pathway and represented a new potential ATC treatment strategy. Although there are no clinical studies, oncolytic virus therapy is a promising treatment for ATC.

### 3.4 Inhibiting recruitment of tumor-associated macrophages (TAMs)

TAMs are mature M2-polarized macrophages derived from blood monocytes and recruited by molecules produced by tumor cells and stromal cells at tumor sites. Increased TAM density has been reported to be associated with a decreased survival rate in TC patients (80). Caillou et al. (81) showed that a large number of TAMs are present in most ATCs. CSF-1 and CCL-2 have chemotactic effects on TAMS; therefore, blocking and targeting the CCL-2/CCR2 and CSF-1/CSF-1R pathways is a promising approach (82). Treatment involving TAMs has been extensively performed in other tumor types, but studies on ATC are relatively lacking. Novel therapeutics are expected to offer better long-term survival for these patients.

## 4 Combination strategies to treat ATC

### 4.1 Combining targeted therapy with chemotherapy or radiotherapy

Recent studies have shown that the combination of targeted drugs and chemotherapy exhibits a synergistic anti-tumor effect (83). In one study, researchers treated ATC cells with lenvatinib and paclitaxel separately or in combination for 72 h and found that lenvatinib enhanced the cell cycle arrest and apoptosis effects of paclitaxel in ATC cells (83). In *in vitro* experiments, researchers established ATC tumor xenografts in nude mice and treated these mice with lenvatinib and paclitaxel, respectively, or in combination. The results showed that the combined treatment had a more significant effect on reducing tumor weight, and lenvatinib enhanced the anti-tumor effect of paclitaxel in ATC (84). In view of this theoretical basis, there are new strategies for targeted drugs with poor monotherapeutic effects. A multicenter, open-label, non-randomized, phase II trial established the safety and tolerability of efatutazone and paclitaxel in anaplastic thyroid cancer, when efatutazone (0.15, 0.3, or 0.5 mg) was administered orally twice daily and then paclitaxel every 3 weeks, respectively (85). Fifteen patients with ATC were enrolled in the study. The median PFS was 48 and 68 days in the 0.15 and 0.3 mg treatment group, respectively. Another open-label, randomized, multicenter study evaluated the safety and efficacy of carboplatin/paclitaxel (CP) with or without fosbretabulin in ATC and concluded that the addition of fosbretabulin to CP did not significantly improve OS (86). In addition, a trial evaluating the efficacy and tolerability of the antifolate agent pemetrexed and the chemotherapy drug paclitaxel in patients with recurrent/advanced follicular, papillary, or anaplastic thyroid cancer was conducted on November 6, 2008, but no results have been released so far (NCT00786552). Currently, there is a study on the treatment of ATC patients with intensity-modulated radiotherapy and paclitaxel, with or without pazopanib (NCT01236547). The preliminary results of this clinical trial show that targeted therapy combined with radiotherapy and chemotherapy does not show a clear therapeutic effect, but the additive effect of its toxicity is not obvious. In summary, targeted therapy combined with chemotherapy has shown positive progress in ATC treatment of anaplastic thyroid cancer.

### 4.2 Combining immunotherapy with chemotherapy or radiotherapy

Some clinical trials have investigated combinations of chemotherapy, radiation, and immunotherapies, especially in combination with immune checkpoint inhibitors, for treatment of patients. Three patients with unresectable tumors were recruited for a phase II study of pembrolizumab combined with ipilimumab, docetaxel, or doxorubicin and volumetric modulated arc therapy (VMAT) as the initial treatment for anaplastic thyroid cancer (87). They had received the study drug (pembrolizumab, 200 mg intravenously) >3 days prior to chemoradiotherapy and then every 3 weeks thereafter until progressive disease, intolerance, or withdrawal of consent. Chemoradiotherapy with docetaxel (20 mg/m^2^) and doxorubicin (20 mg/m^2^) would typically start within 2–4 weeks of surgical resection, as deemed appropriate by treatment providers. For radiation, in the primary setting, VMAT consisted of 66 Gy administered in 33 fractions over 6.5 weeks to all gross diseases in the neck. Early tumor responses were favorable in all three patients, and all three were satisfactorily completed: intended radiotherapy, preceding and radiotherapy-concurrent pembrolizumab, and concurrent chemoradiotherapy. However, all three patients died <6 months (OS) following therapy initiation, prompting study closure. Simultaneously, a phase II trial of atezolizumab in combination with chemotherapy is ongoing (NCT03181100). A trial to test the safety of durvalumab (MEDI4736) and remelimumab in combination with radiotherapy is ongoing (NCT03122496). Few trials have combined immunotherapy with chemoradiotherapy. Although the results of one trial were initially tolerable and effective in terms of disease control in local areas, the disappointing survival results increased the uncertainty about pilot approaches in ATC that merit further pursuit.

### 4.3 Combining immunotherapy with targeted therapy

At present, it has been found that the combined treatment of immunotherapy drugs and targeted therapy drugs may strengthen the antitumor effect of targeted therapy drugs, showing the prospect of combined treatment in ATC.

For patients with ATC, pembrolizumab combined with lenvatinib can effectively delay the progression of the disease, with a median PFS of 16.5 months, but half of the patients experienced adverse effects (88). One study showed that the addition of pembrolizumab in the early stage of progression or KI treatment could also enhance the efficacy of kinase inhibitors to a certain extent (89). There are ongoing experimental studies on this topic. There is a study of whether the standard treatment with dabrafenib and trametinib with the addition of cemiplimab can be an effective method for the treatment of ATC (NCT04238624). To evaluate the combined efficacy of lenvatinib and pembrolizumab in the treatment of ATC, a clinical trial has established a safe and effective treatment for metastatic ATC (NCT04731740). A phase II experiment was conducted to study the effects of pembrolizumab, dabrafenib, and trametinib on the preoperative efficacy of BRAF V600E mutant ATC (NCT04675710). In 2017, Kollipara et al. reported an encouraging case of a 62-year-old man diagnosed with ATC (60). Initially, the patient underwent thyroidectomy and lymph node dissection, followed by chemotherapy. Next-generation sequencing was performed to guide treatment. BRAF and PD-L1 were found to be positive in the tumors, and the patient was treated with vemurafenib (BRAF inhibitor) and nivolumab (human IgG4 anti-monoclonal PD-1 antibody). After 20 months of treatment with nivolumab, metastatic lesions continued to decrease, with complete radiological and clinical remission.

In terms of oncolytic therapy combined with targeted therapy, researchers have made progress in animal experiments. In 2015, Passaro et al. confirmed that PARP inhibition increases dl922‐947 replication and oncolytic activity *in vitro* and *in vivo (* 90). In 2020, Crespo-Rodriguez et al. found that the oncolytic herpes simplex virus (oHSV) in combination with BRAF inhibitors significantly improved survival in ATC mouse models by enhancing immune-mediated antitumor effects, and the combination of PD-1 or CTLA-4 blockade further improved therapeutic efficacy (91).

The use of targeted drugs to enhance the cytocidal activity of NK cells against tumor cells *in vivo* or *in vitro* is also an important research direction. Because indoleamine-2,3-dioxygenase (IDO) can induce the production of kynurenine and reduce the expression of NKG2D and NKP46 receptors in NK cells, the function of NK cells in patients with TC is reduced (92). In 2018, a study showed that prostaglandin E2 (PGE2) produced by TC inhibits the cytolytic activity of NK cells, and ATC cells release more PGE2 than PTC cells (93). Therefore, IDO and PGE2 may be effective targets for enhancing NK cell activity in ATC tissues. Furthermore, IDO1 could play an important role beyond immune regulation, with the potential to influence one-carbon metabolism in cancer cells (94).

In these experimental groups, immunotherapy combined with targeted therapy was more effective than either immunotherapy alone or targeted therapy. However, there are few clinical experiments in this regard, and further research is needed (**Table 3**).

## 5 Conclusion

ATC is a rare and aggressive thyroid cancer that belongs to the thyroid cancer tissue type and has the worst prognosis. Traditional treatments include surgery, radiotherapy, and conventional chemotherapies. However, these treatments are insufficient. Targeted therapy has long been used as a treatment for ATC, while immunotherapy is still in the experimental stage; however, numerous experiments have shown that it can be used as a potential therapy. Studies have found that targeted therapy is beneficial in improving the therapeutic effect and quality of life of patients, but has greater adverse effects. This can be overcome by lowering the dose and using medically assisted therapy for the treatment of adverse symptoms. At the same time, owing to the development of the tumor escape mechanism and the temporary effect, there is rapid development of acquired drug resistance. The response is not durable, and it is necessary to conduct in-depth research on the mechanism of tumor resistance, analyze gene mutations again, and accurately target the treatment site. Currently, one-carbon metabolism has been found to contribute to a variety of downstream pathways known or potentially beneficial for cancer cell survival, and a detailed understanding of it may allow more precise targeting of specific pathways most important for cancer cell survival (95). In immunotherapy, the presence of TAMs, NK cells, and other tumor-infiltrating lymphocytes (TILs) in ATC tissues highlights the correlation of tumor-immune cell interaction (74). Many clinical trials have been conducted on the PD-1 and PD-L1 pathways, which have demonstrated the development of this pathway. Adoptive cell therapy has shown good results in preclinical mouse models of ATC lung metastasis. In addition, oncolytic virus strategies have been shown to play an anti-tumor role through a dual mechanism of selective killing of tumor cells and induction of systemic anti-tumor immunity. Despite major breakthroughs that have been made in clinical practice, immunotherapy remains ineffective for most patients. A variety of unpredictable and carefully managed toxic effects adds to the difficulty of treatment.

Moreover, as the effect of monotherapy for ATC was not satisfactory, researchers considered a multi-drug combination therapy strategy. Toxicity is the biggest limitation of combination immunotherapy, and toxicity is more serious when combining PD-1/PD-L1-targeted drugs and CTLA-4 inhibitory mAb. In addition to toxicity, the appropriate treatment sequence and timing should be considered and selected when designing combination regimens. Further exploration of new drugs and innovative combination strategies are needed to minimize the toxicity of targeted therapies combined with immunotherapy. Predictive biomarkers are urgently needed to guide precise immunotherapy and to explore new combination therapy strategies to harness the immune system to enhance anti-tumor efficacy and deliver optimal treatment to each patient.

In general, effective treatments for ATC are limited, and there is an urgent need to explore new treatments. Although immunotherapy has not yet been approved for ATC, it has been shown to be effective in some malignancies, such as melanoma, non-small cell lung cancer, and leukemia. Compared to targeted therapy, there are fewer studies on immunotherapy, but its successful results should not be ignored. With the development of immunotherapy, it can be hoped and even expected that novel therapeutics arise offering longer term survival for ATC patients. As a promising treatment for ATC, immunotherapy and its combination with targeted therapy should be the focus of future studies.

## Author contributions

XG completed the writing. CH was involved in the design of the manuscripts. YX completed the documentation and figure drawing. XZ were responsible for the revision of the manuscripts. All authors contributed to the article and approved the submitted version.
